# Consumer-grade electroencephalography devices as potential tools for early detection of brain tumors

**DOI:** 10.1186/s12916-020-01889-z

**Published:** 2021-01-22

**Authors:** Nardin Samuel, Emily So, Ugljesa Djuric, Phedias Diamandis

**Affiliations:** 1 Division of Neurosurgery, Department of Surgery, University of Toronto, Toronto, ON Canada; 2 Princess Margaret Cancer Centre, University Health Network, 12-308, 101 College Street, Toronto, ON M5G 1L7 Canada; 3 Department of Laboratory Medicine and Pathobiology, University of Toronto, Toronto, ON M5S 1A8 Canada; 4 Laboratory Medicine Program, University Health Network, 200 Elizabeth Street, Toronto, ON M5G 2C4 Canada; 5 Department of Medical Biophysics, University of Toronto, Toronto, Canada

**Keywords:** Early detection, Brain-machine interface, Primary brain tumors, Non-invasive devices, Electroencephalography, Glioblastoma, Diffuse glioma

## Background

Diffuse gliomas, such as glioblastoma (GBM), represent the most common and aggressive form of brain cancer with an unfortunate dearth of treatment advances despite decades of ongoing research [1]. Perhaps one of the most successful paradigms in cancer management has been early detection programs that aim to dramatically improve outcomes by providing an earlier window for intervention [2]. Indeed, early screening has reduced deaths and improved outcomes of many cancer types (e.g., colon, breast, cervical) when the disease can be detected prior to distant spread [3]. In support of this paradigm in neuro-oncology, the achievement of gross total surgical resection in younger patients with GBM shows favorable outcomes compared to instances where only incomplete resection is possible (37.3 vs. 16.5 months) [4]. Therefore, if gliomas could be detected earlier in their evolution, while remaining potentially more localized, it could also offer the possibility to better maximize the extent of safe surgical resection and favorable outcomes.

Despite this prospect, the aggressive biology and low incidence of gliomas make them poor candidates for conventional screening strategies. Case reports of serial neuro-imaging studies of GBM, with scans taken as few as 68 days apart, show that even small cortical lesions can rapidly evolve into established disease within a very short clinical time frame (< 3 months) [5]. This short interval in evolution means that potentially effective screening programs would require assessments every 2–3 months to allow detection of incipient lesions. This, combined with its low incidence (3–4/100,000 people), makes radiographic or other annually administered screening approaches for gliomas impractical.

## Brain wearables as potential tools for early detection

**Fig. 1:**
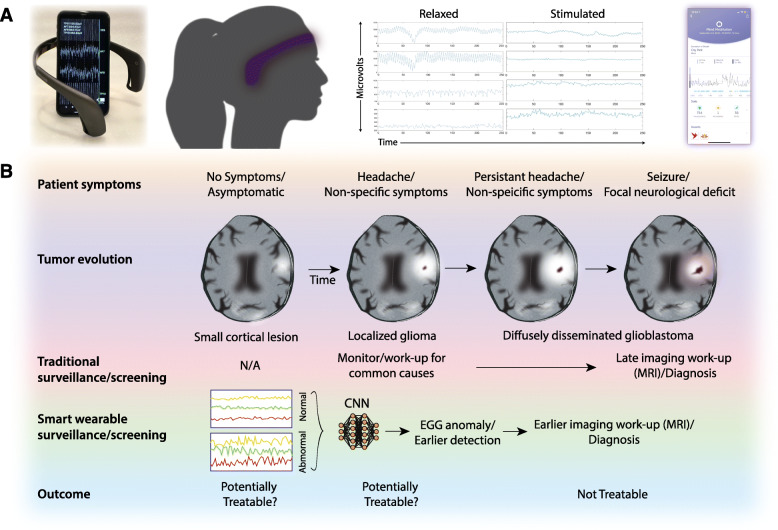
Brain wearables as agents to monitor brain health. **a** Photo of the Muse® 2 headband transmitting electrical brain activity to a smartphone. This particular model is worn over the forehead (2 frontal EEG electrodes) and behind the ears (2 temporal EEG electrodes) and also contains sensors to measure heart rate and head movement. Example of EGG data captured by device across different mental states (e.g., active vs. relaxed). These pattern changes can be interpreted using machine learning to provide dynamic real-time feedback to users through custom smartphone applications (e.g., quality of meditative states). Newer portable EEG devices are now adding additional electrodes to expand spatial resolution and applications. **b** The non-specific and relatively late-onset symptoms of brain tumors have not allowed for early and population-wide screening. Continuous monitoring of brain activity with smart wearables, when coupled with deep learning algorithms (e.g., convolutional neural network (CNN)), could however allow for earlier detection of pathology at a population level. The recent favorable outcomes seen with aggressive surgical resection for gliomas provide optimism that early intervention may allow for more complete removal of disease and improved outcomes

Consequently, when conceptualizing an effective screening tool for gliomas, brain health changes would need to be monitored over short intervals (days to weeks) using tools that could be practically applied to the general population. Herein lies the immense potential of using emerging non-invasive electroencephalography (EEG)-based biotracking devices to serve as agents that gather continuous health data from our nervous system (Fig. 1a). These consumer-grade devices, often worn as headbands, have 4–14 dry scalp electrodes and can be set up in 1–2 min and immediately begin collecting real-time electrical signals focally generated during different forms of brain activity. As 68–85% of brain tumor patients have abnormal EEG profiles at diagnosis [6], continuous monitoring of subtle changes in the brain’s electrical ground state by these devices may afford detection and differentiation between different neuropathologies, including brain tumors, earlier in their pre-symptomatic state (Fig. 1b). Optimistically towards this prospect, there has already been a willingness by hundreds of millions of citizens to adopt other “smart devices” (e.g., FitBit/Apple Watch) allowing for continuous recordings of electrocardiogram tracings. In addition to their motivational benefits, the ability to track and detect abnormal heart rhythms across a large fraction of the asymptomatic population is already being explored as an early detection tool for cardiovascular causes of stroke [7]. More recently, there has been significant progress in the development of cost-effective portable EEG wearables (~ $250 USD) capable of monitoring brain activity in real time with qualities beginning to rival traditional medical-grade devices [8]. Using machine learning, such devices are now in routine use to autonomously detect changes in mental states and provide feedback for mediation, relaxation, and sleep health. Given their scalability, these devices are now also being utilized for population-level analyses of brain activity across a wide array of physiological and pathological states [8]. If indeed adoption of personal EEG devices also soon becomes widespread, early detection of brain tumors may be one intriguing application. Recent demonstrations that brain tumors integrate and participate in native synaptic circuits provide biological support that electrical perturbations, including disorganized rhythms and/or attenuated background activity, may be underappreciated early biomarkers of these neoplasms [9].

## Potential limitations

With these exciting prospects in mind, several potential limitations at present are worth highlighting. Current EEG wearables, designed for niche application (e.g., mediation), only require a handful of sensors for their intended uses. The optimal quantity and spatial distribution of sensors for effective screening of central nervous system pathologies therefore still need to be defined. Additionally, the amount and timing of daily wear needed to detect subtle baseline changes would also need optimization and balance with enthusiasm to wear such devices. Despite these current unknowns, rapid and widespread acceptance of other smart wearables offers encouraging insights. For example, despite only debuting in 2015, Apple shipped > 40 million smartwatches in 2018 with the global wearable market expected to grow to > 279 million users by 2023. This estimate of the wearable market alone could allow the detection of tens of thousands of brain tumors annually! With increasing possibilities and interest at the intersection between artificial intelligence and brain-machine interfaces (recently revitalized by Elon Musk’s Neuralink), such devices may soon even become essential tools to intellectually compete in society. It is therefore possible that consumer demand for brain wearables may quickly mirror the rapid growth of their hardware manufacturers and extend far beyond current niche applications and existing smartwatch demands [10]. Lastly, like any screening tool, a beneficial balance between sensitivity and specificity, along with proven beneficial outcomes following early detection and treatment, will still need to be formally demonstrated [7].

The ability of these devices to provide continuous longitudinal and personalized data, along with the advent of modern deep learning computational tools, could provide new solutions for the early detection and differentiation of various nervous system pathologies. Large-scale detection of incipient tumor formation could also provide new epidemiological insights of unappreciated risk factors and mechanisms of gliomagenesis. Deployment of these devices to existing brain tumor patients could also help monitor disease recurrence and understand treatment-related neurocognitive sequalae.

## Conclusions

Brain tumors such as GBM carry a dismal prognosis which has challenged conventional treatment paradigms for decades. Conceptualizing novel early detection-based approaches for these tumors may provide success in a patient population where conventional late-stage diagnosis and treatment have failed. While the role of early detection in the management of brain tumors is still unclear, recent innovations in personal brain wearables, artificial intelligence, and evidence for the benefits of aggressive surgery offer new opportunities to refine our clinical approach to these challenging clinical entities.

## Data Availability

Not applicable.
